# Brain–machine interface based on deep learning to control asynchronously a lower-limb robotic exoskeleton: a case-of-study

**DOI:** 10.1186/s12984-024-01342-9

**Published:** 2024-04-05

**Authors:** Laura Ferrero, Paula Soriano-Segura, Jacobo Navarro, Oscar Jones, Mario Ortiz, Eduardo Iáñez, José M. Azorín, José L. Contreras-Vidal

**Affiliations:** 1 Brain-Machine Interface Systems Lab, Miguel Hernández University of Elche, Elche, Spain; 2 Instituto de Investigación en Ingeniería de Elche-I3E, Miguel Hernández University of Elche, Elche, Spain; 3 International Affiliate NSF IUCRC BRAIN Site, Miguel Hernández University of Elche, Elche, Spain; 4 NSF IUCRC BRAIN, University of Houston, Houston, USA; 5 International Affiliate NSF IUCRC BRAIN Site, Tecnológico de Monterrey, Monterrey, Mexico; 6 Non-Invasive Brain Machine Interface Systems, University of Houston, Houston, TX USA; 7 Valencian Graduate School and Research Network of Artificial Intelligence-valgrAI, Valencia, Spain

**Keywords:** Brain–machine interface, EEG, Exoskeleton, Deep learning, Transfer learning

## Abstract

**Background:**

This research focused on the development of a motor imagery (MI) based brain–machine interface (BMI) using deep learning algorithms to control a lower-limb robotic exoskeleton. The study aimed to overcome the limitations of traditional BMI approaches by leveraging the advantages of deep learning, such as automated feature extraction and transfer learning. The experimental protocol to evaluate the BMI was designed as asynchronous, allowing subjects to perform mental tasks at their own will.

**Methods:**

A total of five healthy able-bodied subjects were enrolled in this study to participate in a series of experimental sessions. The brain signals from two of these sessions were used to develop a generic deep learning model through transfer learning. Subsequently, this model was fine-tuned during the remaining sessions and subjected to evaluation. Three distinct deep learning approaches were compared: one that did not undergo fine-tuning, another that fine-tuned all layers of the model, and a third one that fine-tuned only the last three layers. The evaluation phase involved the exclusive closed-loop control of the exoskeleton device by the participants’ neural activity using the second deep learning approach for the decoding.

**Results:**

The three deep learning approaches were assessed in comparison to an approach based on spatial features that was trained for each subject and experimental session, demonstrating their superior performance. Interestingly, the deep learning approach without fine-tuning achieved comparable performance to the features-based approach, indicating that a generic model trained on data from different individuals and previous sessions can yield similar efficacy. Among the three deep learning approaches compared, fine-tuning all layer weights demonstrated the highest performance.

**Conclusion:**

This research represents an initial stride toward future calibration-free methods. Despite the efforts to diminish calibration time by leveraging data from other subjects, complete elimination proved unattainable. The study’s discoveries hold notable significance for advancing calibration-free approaches, offering the promise of minimizing the need for training trials. Furthermore, the experimental evaluation protocol employed in this study aimed to replicate real-life scenarios, granting participants a higher degree of autonomy in decision-making regarding actions such as walking or stopping gait.

**Supplementary Information:**

The online version contains supplementary material available at 10.1186/s12984-024-01342-9.

## Background

Brain–machine interfaces (BMIs) have recently emerged as promising rehabilitation tools for promoting recovery of lost motor function. By enabling direct communication between the brain and an external device such as a robotic exoskeleton, BMIs offer a novel approach to rehabilitation. Subjects are required to engage in specific mental practices that translate into concrete actions in the output device. One such practice is motor imagery (MI), which involves the imagination of a given movement without actually executing it. When used to generate a movement-associated stimulus that is provided by the robotic exoskeleton, MI offers an effective strategy for facilitating motor recovery through enhancing the principles of neural plasticity [1].

There are several challenges BMI must face, one of which is related to the recording system used to capture brain activity [2]. While functional magnetic resonance imaging (fMRI) can serve as a recording system, portable systems such as electroencephalography (EEG) or electrocorticography (ECoG) are preferred for rehabilitation applications [3]. Differences between EEG and ECoG rely on if the electrodes used to measure the brain signals are non-invasive (EEG) or invasive (ECoG). Consequently, most BMIs are based on EEG [4]. However, EEG has its own limitations. First, the signal-to-noise ratio is lower than the other two systems due to the susceptibility of the signal to various artifacts such as movement, sweating or external electromagnetic fields. Second, EEG signals are non-stationary, meaning that their properties can differ significantly between different rehabilitation sessions, or even within the same session [2, 5].

On the other hand, all EEG-based BMIs require calibration, during which the system learns to discriminate brain activity through training under different mental strategies. Given the non-stationary nature of the EEG signals, this process must be performed for each subject and experimental session, rendering it time-consuming and potentially fatiguing for subjects [2].

Given the limitations of EEG-based BMI, processing algorithms have been developed to extract generic features capable of discriminating different brain patterns across all subjects. Traditional algorithms rely on manually designed temporal, spectral, or spatial features [6]. When considering temporal features, various metrics can be computed to provide information about the signal, such as the mean, median, standard deviation, or kurtosis [7]. Other time domain features based on amplitude modulation (AM) or the readiness potential have been successfully used a features [6, 8, 9]. Along with temporal features, spectral features have also been extensively studied in MI-based BMIs. Researchers have utilized power spectral density (PSD) as a discriminative feature in frequency ranges associated with motor planning and execution, such as theta, alpha, and beta [10, 11]. Various alternatives based on PSD have been proposed, including computing a relative PSD of the MI period with respect to a baseline [6, 12]. Additionally, some analyses have represented signals as a time-frequency resolution of magnitude and phase, rather than computing a single PSD over a period of time. Examples of such methods include the Stockwell transform [13, 14] and the Wavelet transform [2]. In addition to spectral features, two state-of-the-art methodologies in MI-based BMI rely on spatial features: common spatial patterns (CSP) [15] and Riemannian manifold [16]. Both methods measure how each channel is related to the rest by computing correlation or covariance matrices respectively.

Deep learning has emerged as a promising technique in various fields, including BMI systems, as it eliminates the need for manual computation of features [17]. Many previous works have utilized deep learning approaches with raw or normalized EEG signals as input, which were band-pass filtered to the frequencies of interest such as alpha or beta [17–19]. Alternatively, some studies have applied Wavelet transform or CSP prior to using neural networks [20], or have used time-domain AM EEG features to train deep network architectures [8]. When it comes to the architecture of the deep learning model, studies that focused on MI as the mental strategy have preferred convolutional frameworks to capture spatial relationships among different brain areas [17, 21–23]. Another advantage of deep learning is the ability to perform transfer learning, where models can be trained with data from different domains and then fine-tuned for the desired one [22, 24, 25]. While transfer learning can also be used with traditional methodologies, limitations arise due to differences in manually extracted features across different domains. While deep learning presents numerous advantages over feature-based methodologies, its application in BMI systems with real-time control of robotic exoskeletons remains limited. As elucidated in the literature [26], deep learning techniques have primarily been explored in offline BMI scenarios due to their prolonged training durations. The imperative for practical online BMI applications demands classifiers capable of rapid training, ideally within a few minutes, to facilitate real-world deployment. However, the computational complexity associated with deep learning poses a significant challenge. To address this, an alternative approach, as proposed in [26], involves the exploration of systems that obviate the need for subject-specific training.

In this study, we focused on developing a MI-based BMI using deep learning algorithms to control a lower-limb robotic exoskeleton. When designing a protocol for such a system, two alternatives have been proposed in the literature: synchronous and asynchronous. In synchronous BMIs, the researcher informs the subject when to perform each mental task and when to expect specific actions in the exoskeleton conditioning the subject. In contrast, asynchronous BMI is considered more natural as the subject decides when to perform each mental task without time constraints and any external cue. However, evaluating the efficacy of the system is challenging in an asynchronous BMI [27]. The main contributions of this study are:

- A BMI design based on neural networks that applies transfer learning, allowing to combine data from different subjects and sessions to train the model and reduce the calibration time. This approach was compared against a CSP-based approach that uses conventional calibration.
- A proposal for an evaluation protocol that simulates a real-life scenario where participants need to travel a path with a series of stops, validating the performance through an objective oriented assessment instead of artificial external cues limited by time.
- An evaluation of the system with 5 participants.

## Methods

### Subjects

The experiments were conducted with the participation of five healthy subjects who did not report any known diseases or movement impairment and had no prior experience with BMI systems (mean age, 22.6 ± 3.05). Prior to the experiments, the participants were informed about the study and provided written informed consent. All the procedures were approved by the Institutional Review Board of the University of Houston, TX (USA), with study ID: STUDY00003848.

### Equipment

During the experiments, EEG signals were recorded using 32 wet electrodes positioned over an actiCAP (Brain Products GmbH, Germany). Two additional electrodes, serving as ground and reference, were located on the ear lobes. Electrodes were placed following the 10–10 distribution, with four electrodes used for recording electrooculography (EOG), arranged in a cross shape with respect to the eye with the vertical ones around the left eye. The data were wirelessly transmitted using a WiFi MOVE unit (Brain Products GmbH, Germany) and amplified with BrainAmpDC (Brain Products GmbH, Germany).

**Fig. 1:**
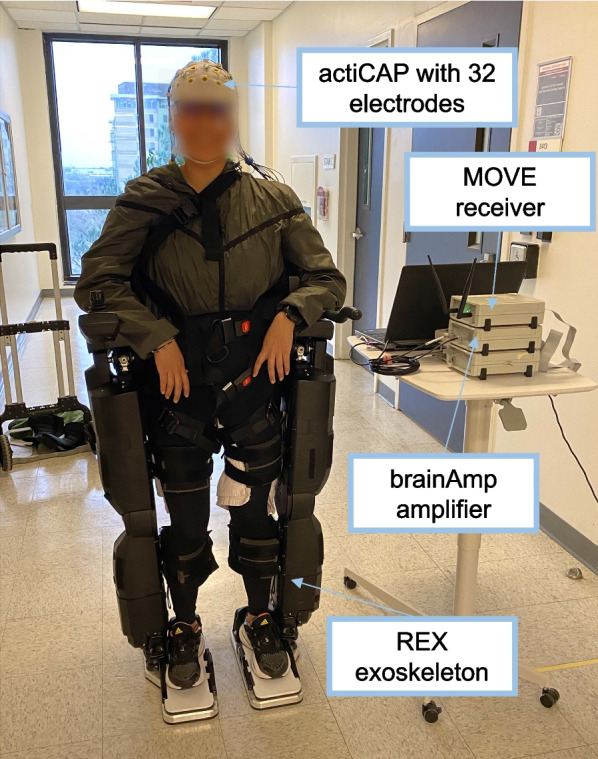
Equipment employed in the experiments

The REX exoskeleton (Rex Bionics, New Zealand) was utilized for the experiments. This exoskeleton is capable of independently supporting both itself and the weight of the subject, making it suitable for individuals with complete spinal cord injury. It is comprised of powered hip, knee and ankle joints (bilaterally). This self-standing exoskeleton does not require crutches and can be controlled by high-level commands sent via Bluetooth to initiate or stop the gait. Real-time feedback on the exoskeleton status was also provided during the experiments. Figure 1 shows the equipment employed in the experiments.

### Experimental protocol

**Fig. 2:**
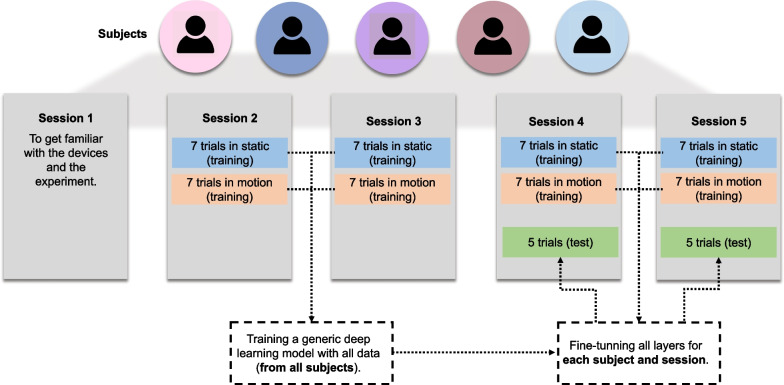
Five subjects participated in five experimental sessions

In the study, five experimental sessions were conducted by the participants to test a lower-limb exoskeleton (REX) system controlled by a BMI as shown in Fig. 2.

#### First session

In the first session, participants were given an overview of the experimental protocol and asked to complete a consent form. An initial assessment was conducted to ensure that participants met the inclusion/exclusion criteria, see Additional file 1. They were also asked general questions about their current physical state. If they were eligible to participate, baseline measurements were taken to properly set up the brain cap and exoskeleton, including foot length and width, weight, height, lower limb lengths, and head size. Participants had the opportunity to gain some experience with the exoskeleton. No EEG recordings were accomplished during this first session.

Afterwards, the concept of kinesthesic and visual motor imagery were explained [28]. In addition, they received the Motor Imagery Questionnaire-3 (MIQ-3) to complete at home. Two different versions, Spanish or English, were provided depending on the subjects’ mother tongue [29, 30]. The MIQ-3 is a 12-item questionnaire designed to evaluate the individual’s capacity to mentally visualize four specific movements using internal visual imagery, external visual imagery, and kinesthetic imagery. Kinesthetic motor imagery refers to the cognitive capacity to mentally simulate the execution of a physical action by generating a vivid perception of the muscular contractions and sensations that accompany the actual movement. In contrast, visual motor imagery involves the ability to create a mental representation of the desired movement. During the following sessions, participants were instructed to perform only kinesthetic motor imagery since it produces more similar brain patterns as motor execution and therefore, it promotes mechanisms of neuroplasticity that induces motor rehabilitation [31].

#### Second and third session

In the second and third session, participants wore the EEG equipment and lower-limb exoskeleton and walked with it for 30 min while being commanded by an external operator/researcher before the real experiment began. This preliminary phase aimed to acquaint participants with the device prior to commencing the actual experimental tasks. The operator sent commands from a computer to the exoskeleton via Bluetooth to start or stop the gait at certain periods, with participants being given an acoustic cue beforehand.

**Fig. 3:**
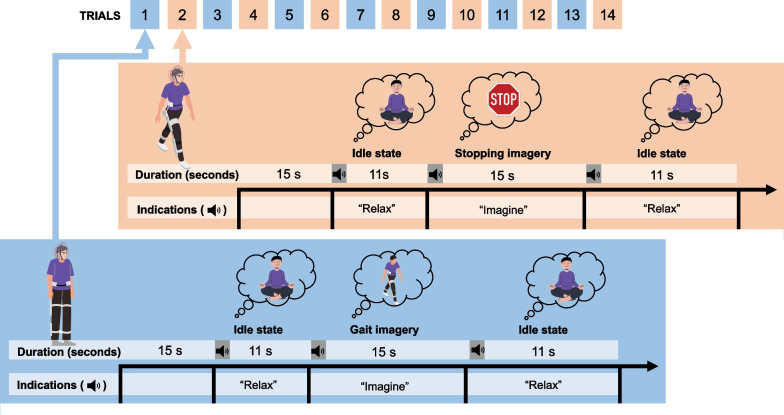
BMI calibration. It involved the training phase, during which participants completed a total of 14 trials involving specific mental tasks. Half of these trials were conducted under full static conditions (blue), where participants stood still with the exoskeleton, while the other half involved walking assisted by the exoskeleton during the whole trial (orange). The trials conducted under static and motion conditions followed a similar structure. Each trial started with a 15-s period to allow the convergence of the denoising algorithms. Subsequently, an acoustic cue signaled the initiation of the idle state, during which participants were instructed to relax. Following this, another cue indicated the onset of the motor imagery period. Notably, the motor imagery task differed between static and motion trials. In the static trials, participants were instructed to imagine the act of walking, whereas in the motion trials, the task involved imagining the action of stopping the gait. Specifically, this stopping action was defined as bringing the legs together after completing a step

After becoming familiar with the device, training with the BMI and the lower-limb exoskeleton began, which is also referred as calibration. Participants performed 14 trials with the exoskeleton in open-loop control, during which they engaged in a series of mental practices including idle state and kinesthetic motor imagery. The sequence of tasks is shown in Fig. 3.

During half of the trials, participants stood still with the exoskeleton, and during the other half, they walked. The lower-limb exoskeleton was controlled the whole time by the predefined open-loop controlled periods. These procedural steps were implemented to facilitate the development of a dual-state BMI, specifically comprising two distinct models: Static and Motion, which were then used in the closed-loop control phase.

#### Fourth and fifth session

In the fourth and fifth sessions, participants were first fitted with EEG equipment and lower-limb exoskeleton. They then walked with the exoskeleton for a period of 30 min, which was controlled by an external operator/researcher before the actual experiment began.

Following this, the participants underwent training with a BMI in the same manner as in the second and third sessions. However, after the training, the BMI was updated with data specific to each participant and session, and it was then tested in closed-loop control as shown in Fig. 2.

**Fig. 4:**
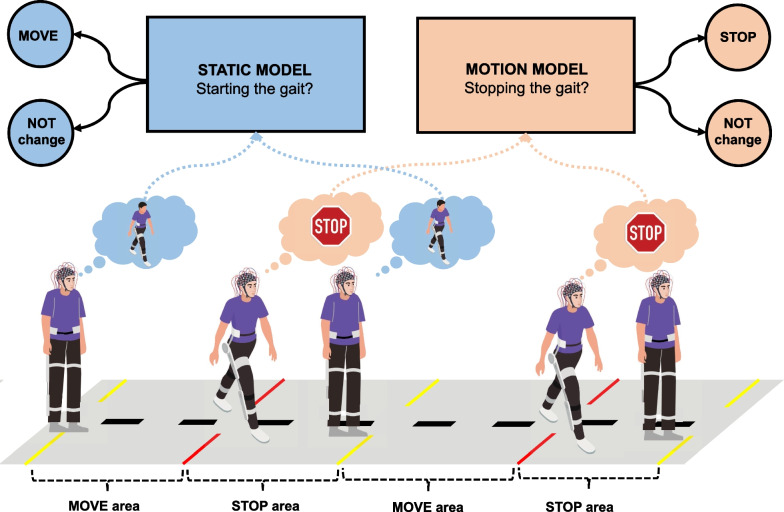
Asynchronous closed-loop control. During this phase, participants engaged in five trials where they utilized their thoughts to control the lower-limb exoskeleton. The experimental setup involved navigating through a pathway that was divided into distinct regions: MOVE areas marked by yellow lines and STOP areas demarcated by red lines. Within the MOVE areas, participants were required to engage in motor imagery of the gait until a command was sent to the exoskeleton, initiating the walking motion. To maintain the gait, participants were instructed to maintain an idle state until they reached the STOP area. Upon entering the STOP area, participants were tasked with performing a single stop. This involved mentally imagining the movement of stopping the gait. Participants were required to sustain this mental task until a command was issued to the device or until they exited the STOP area and reentered a MOVE area. Failure to execute a stop within the designated STOP area constituted an unsuccessful attempt

To test the BMI, participants walked along a straight path that had five lines marked on the floor. The yellow lines marked the areas in which the subject should begin walking, while the red lines marked the areas in which they should stop. Participants had to perform various mental tasks to make the exoskeleton move or stop. They were trained during the previous sessions to imagine themselves during two different classes (MI and idle state) and in two different states (Static and Motion): static motor imagination for starting the gait vs. static in an idle state to remain standing still; and motion in an idle state to continue walking vs. motion motor imagination for stopping the gait. A diagram of the path is shown in Fig. 4. They performed five test trials and in each of them they had to go through the whole move/stop areas path. This protocol was designed as an asynchronous control, so subjects decided when to begin each mental task trying to reach the different stop areas keeping the exoskeleton in motion up to them.

As mentioned above, two models were trained with data from training phase: Static model was trained only with the trials in which subjects were standing still, and Motion model was trained only with trials in which subjects were walking assisted by the exoskeleton. They were utilized for the control process as a dual-state machine. The Static model had the purpose of preserving the exoskeleton in a stationary position and detecting the initiation of gait. However, once the exoskeleton started its movement, the control mechanism of BMI shifted to the Motion model. This model effectively controlled the continuous motion of the exoskeleton until a desire to halt its progression was detected. Upon such detection, the control model was switched to the Static model again. Figure 4 shows a schema of this dual-state control.

### Brain–machine interface

#### Deep learning

EEGNet [32] was used in the experiments. This network combines the principles of temporal, frequency and spatial features that were manually computed in traditional approaches. This framework starts with a temporal convolution to learn specific frequency filters that highlight relevant brain rhythms. It is followed by a depthwise convolution in spatial dimension that learns a spatial filter for each filtered signal from the previous layer. Finally, the separable convolution is a combination of a depthwise convolution that learns a temporal summary from each spatially filtered signal from the previous step, and a pointwise convolution that combines all features in the most discriminant way. This network was preferred for this experiment due to its relatively low number of trainable parameters as compared to other frameworks present in the literature, such as DeepConvnet [18]. The network hyper-parameters are shown in Table 1.

**Table 1:** EEGNet hyper-parameters

Learning rate	0.001
Batch size	32
Epochs	80
Dropout rate	0.4

Two networks were trained, one with static trials (Static model) and one with trials in motion (Motion model). The Static model’s input data consisted of 2 s epochs of the pre-processed EEG signals of 27 channels sampled at 200 Hz. Each epoch was shifted at a 0.5 s pace, so they were overlapped 1.5 s. Pre-processing involved applying a Notch filter at 60 Hz to remove the contribution of the power line and a high-pass filter at 0.1 Hz to reduce DC offset. A denoising algorithm was employed using the four EOG channels to estimate the contribution to each EEG channel and mitigate the artifact contribution [33]. The following step was to apply a common average reference (CAR) spatial filter [34], to enhance the activity of each electrode by subtracting the mean from all of them for every time point. Finally, a band-pass filter was applied between 8 and 40 Hz to focus on alpha, beta and low gamma rhythms [19].

For the Motion model, input data were pre-processed in the same window size and sliding window, but with a slightly different method. The first steps till CAR spatial filter were the same. However, the following steps were a band-pass filter between 1 and 100 Hz [17], and signals were normalized [14]. The selection of pre-processing approaches was guided by the findings of our prior research [35], which identified the approach that yielded the most favorable outcomes.

In this study, we conducted a comparative analysis of three distinct training sub-approaches for both static and motion networks. The selection of the optimal approach for closed-loop control was based on the results obtained from an open-loop evaluation pseudo-online, which means they were evaluated post hoc after the completion of all the sessions simulating a real-time prediction system. The three sub-approaches investigated were: (1) a generic model, (2) a generic model fine-tuned to individual subject and session data, and (3) a generic model fine-tuned to individual subject and session data with a focus on the last three layers.

**Fig. 5:**
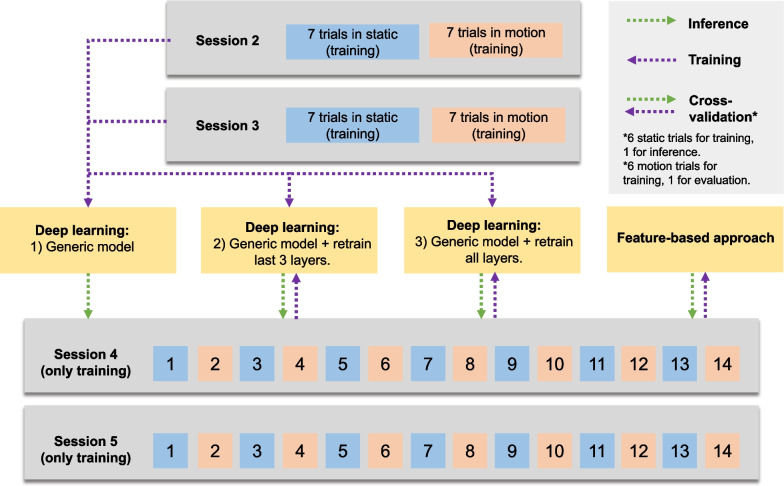
Visual representation of the training and evaluation procedures

To elucidate the training and evaluation procedures, Fig. 5 provides a visual representation of the following:

1. **Deep learning with generic model:**
  The model was trained with training data from sessions 2 and 3 from all subjects.
  Evaluation was performed using data from sessions 4 and 5.
2. **Deep learning with generic model and fine-tuning:**
  The generic model was initially trained with data from sessions 2 and 3 from all subjects.
  In session 4, fine-tuning involved 12 training trials and 2 evaluation trials. This process was iterated, with each trial serving as evaluation once and remaining ones for fine-tuning parameters (static model: 6 fine-tuning and 1 evaluation, motion model: 6 fine-tuning and 1 evaluation).
  Similar procedures were applied in session 5. Therefore, the generic model was adapted to each subject and session.
3. **Deep learning with generic model and fine-tuning last three layers only:**
  Similar to the previous model, training and evaluation were conducted using data from sessions 2, 3, 4, and 5.
  However, fine-tuning focused exclusively on the last three layers of the generic model.

The second alternative showed the highest results (it can be seen in “Results”), and thus these models were used in the closed-loop evaluation during the fourth and fifth sessions.

#### Features-based approach

The three deep learning sub-approaches aforementioned were compared against a baseline approach that is feature-based and commonly used for BMI [36–38]. Pre-processing of signals was performed differently for the features-based approach than for neural networks. Firstly, a Notch filter was applied at 60 Hz to remove power line noise, followed by a high-pass filter at 0.1 Hz to reduce the DC offset. The same EOG denoising algorithm employed in deep learning was then applied, as previously described [33]. Subsequently, four band-pass filters were applied at 5–10, 10–15, 15–20 and 20–25 Hz, consistent with our previous works [36, 37]. The next step involved computing CSP for each frequency band. The goal of CSP was to calculate spatial filters that linearly transform the signal from each channel to maximize differences between two mental tasks, in this case, between MI of gait and idle state for both models, static and motion. The signals from 27 electrodes were filtered, and only the eight most discriminant new components were selected as features. The log-variance was computed for all of them, resulting in a vector of 32 features (8 × 4 frequency bands). Linear discriminant analysis (LDA) was trained with these features to distinguish between the two classes: MI and idle state.

The training and evaluation of this approach are illustrated in Fig. 5. In session 4, 12 trials were used to train the model and 2 for evaluation. This process was repeated, using all trials for evaluation once (static model: 6 training and 1 evaluation, motion model: 6 training and 1 evaluation). The same procedure was applied in session 5. Data from sessions 2 and 3 were not included in training the model because CSP has previously shown poor generalization and higher performance when trained with each subject and session’s data [38, 39].

### Evaluation

The efficacy of the BMI was evaluated using a set of defined metrics. The evaluation encompassed both training data and closed-loop trials, providing a comprehensive assessment of the system capabilities. The following metrics were employed:

1. Evaluation of training data (open-loop pseudo-online): cross-validation was performed and the accuracy was measured as the percentage of epochs with correct classification during trials, both in static and motion conditions.
2. Evaluation of closed-loop trials:
  Average time to Start: This metric quantified the duration, measured in seconds, that subjects required to send a START command to the exoskeleton while in a static state and performing MI. Notice that as the starting moment of the imagination is marked by the subject will, a high value does not mean a bad evaluation. However, an excessive time could be considered as a timeout.
  Average time to Stop: This metric measures the time, in seconds, participants took to issue a STOP command to the exoskeleton while in motion, providing insights into the promptness of their response.
  Timeout: In cases where participants were unable to send a START command within 60 s, the trial was considered a timeout. Additionally, if the subject made eight attempts to activate the exoskeleton but failed to reach the end of the path, it was also categorized as a timeout. This metric captured the number of trials that resulted in a timeout.
  Accuracy to Start (%): This percentage reflected the frequency at which subjects successfully sent a START command without experiencing a timeout. It indicated the proficiency of subjects in initiating the desired actions within the given time-frame.
  Accuracy to Stop (%): This metric measured the success rate of subjects in stopping the exoskeleton within the designated STOP areas along the five testing trials. The presence of two STOP areas allowed for the calculation of a percentage. If subjects managed to make a stop in both areas, it would be considered 100% accuracy, while a single stop corresponded to a 50% accuracy for the trial. A value of 0% indicated that the exoskeleton failed to stop at all.
  Ratio of Stops (%): This ratio provided insights into the quality of the stops made by the device. It measured the proportion of successful stops with respect to the total number of stops performed.

## Results

Participants engaged in a total of five sessions, during which training with the BMI was conducted in all sessions except for the initial one, which solely served the purpose of familiarizing participants with the system. The evaluation, i.e., closed-loop control, was only performed in fourth and fifth sessions. The data acquired from the second and third sessions were utilized to train a generic model that was evaluated in the last two sessions. As outlined in the preceding section, four distinct evaluation approaches were employed, three deep learning methods and a features-based approach.

To evaluate the calibration data, the training data from the fourth and fifth sessions were utilized to infer predictions of either idle state or MI. In the first deep learning approach, the trained generic model was directly employed for predictions (1). In the second and third approaches, a fine-tuning was performed updating the model with the information derived from the fourth and fifth sessions. However, the adaptation process varied between the two cases. In one scenario, all layers of the network were subjected to fine-tuning (2). In the other case, only the last three layers of the network underwent retraining (3). On the other hand, the features-based model was only trained with data from the fourth or fifth sessions respectively without using data from the previous sessions or other subjects. The initial approach was assessed by extrapolating predictions in training trials from fourth and fifth sessions at intervals of 0.5 s (1). The last three approaches, deep learning with fine-tuning (2, 3) and the features-based model, were evaluated using cross-validation. For instance, if the fourth session comprised seven static training trials, six of them were utilized for fine-tuning or training the features-based model from scratch, while the remaining trial was reserved for evaluation. This procedure was repeated, utilizing each trial once for evaluation purposes. This comprehensive process was carried out separately for static and motion trials and predictions were also given every 0.5 s.

Table 2 shows the results of the four approaches for each participant and as the average of fourth and fifth sessions. The deep learning approach with a generic model and fine-tuning all layers (2) was the one that showed the highest results.

**Table 2:** Results from training data of fourth and fifth sessions

			A01	A02	A03	A04	A05	Avg.
*Static*	DL	Re-train all	50.8	55.9	67.6	76.5	69.9	64.1 ± 10.6
		Re-train last 3	43.5	55.2	64.8	74.2	68.5	61.3 ± 12.1
		No re-train	51.7	53.8	62.9	70.5	65.1	60.8 ± 7.9
		FA	55.4	60.1	57.3	65.9	64.5	60.6 ± 4.6
*Motion*	DL	Re-train all	52.9	66.2	61.7	65.2	54.2	60.1 ± 6.2
		Re-train last 3	49.2	59.7	50.6	60.1	49.0	53.7 ± 5.7
		No re-train	53.3	61.0	52.4	60.6	51.7	55.8 ± 4.6
		FA	56.5	59.8	55.5	57.8	55.1	56.9 ± 1.9

*DL* deep learning, *FA* features-based approach

Statistical differences among subjects were assessed using a one-way analysis of variance (ANOVA) with the model $accuracy \sim \text {subject}$ for both static and motion data. The normality assumption was examined using the Shapiro–Wilk test, revealing no evidence of non-normality for static data (*A*01: *W* = 0.982, *p* > 0.01; *A*02: *W* = 0.970, *p* > 0.01; *A*03: *W* = 0.982, *p* > 0.01; *A*04: *W* = 0.982, *p* > 0.01; *A*05: *W* = 0.971, *p* > 0.01) and motion data (4*A*01: *W* = 0.962, *p* > 0.01; *A*02: *W* = 0.984, *p* > 0.01; *A*03: *W* = 0.945, *p* > 0.01; *A*04: *W* = 0.960, *p* > 0.01; *A*05: *W* = 0.964, *p* > 0.01). The assumption of homoscedasticity was evaluated using the Breusch-Pagan test, indicating homoscedasticity for static trials (*BP*(4) = 2.9483, *p* > 0.01) but non-homoscedasticity for motion trials (*BP*(4) = 16.78, *p* < 0.01). Consequently, static data underwent the original ANOVA test, revealing significant differences among subjects ($F(4,275) = 42.58, p<0.01$), while motion data were subjected to the Kruskal–Wallis non-parametric test, indicating significant differences among subjects ($\chi ^2(4)= 36.64, p<0.01$).

Differences among BMI methodologies and sessions were further analyzed using a two-way repeated measures ANOVA: $accuracy \sim \text {methodology} \times \text {session}$. The first hypothesis examined whether distinct BMI methodologies exhibited significant performance differences. The second focused on the impact of practice on system efficacy, assessing if the fifth session significantly outperformed the fourth. The third explored synergy between methodology and session, investigating if the classifier achieving the highest results varied across sessions. The Shapiro–Wilk test did not show evidence of non-normality for any group, with no significant outliers detected, and Mauchly’s Test of Sphericity confirmed equal variances of group differences. Results from static trials showed no significant differences in terms of methodology ($F(3,102)= 2.529, p>0.01$), session ($F(1,34)= 1.307, p>0.01$), and the interaction between both was non-significant ($F(3,102)= 2.911, p>0.01$). In the context of motion trials, distinctions in methodology yielded statistical significance ($F(3,102)= 6.298, p<0.01$), while disparities in session outcomes were not significant ($F(1,34)= 0.007, p>0.01$). Additionally, the interaction between methodology and session exhibited non-significance ($F(3,102)= 1.210, p>0.01$).

**Fig. 6:**
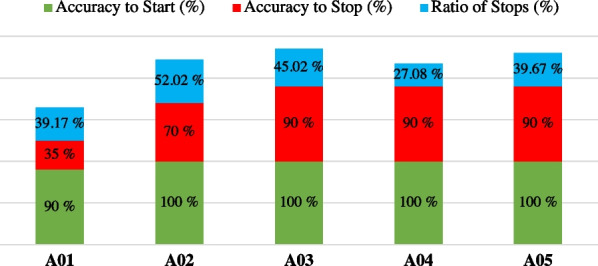
Results from closed-loop control. Subjects were tasked with traversing a designated pathway, requiring them to engage in specific mental imagery practices. To initiate the gait, participants were instructed to imagine themselves walking. Subsequently, they were required to maintain an idle state to sustain the walking motion. When it came to halting the gait, subjects were instructed to imagine the act of stopping. Metrics represent the successful number of activations and stops

During the fourth and fifth sessions, the closed-loop control approach was employed to assess the performance of BMI. Participants utilized their mental practices to elicit desired changes in the robotic exoskeleton, while navigating through a path designed to simulate real-life scenarios. Since the deep learning approach that involved a generic model with fine-tuning all the layers (2) was the one that showed the highest results in open-loop trials, it was used in this closed-loop control phase. The results of these experiments are summarized in Table 3 and Fig. 6.

**Table 3:** Results from closed-loop control of fourth and fifth sessions (averaged across trials)

	Time start (s)	Time stop (s)	Timeout (no)	Acc. start (%)	Acc. stop (%)	Ratio stops (%)
*A01*						
Session 4	7.5 ± 3.8	31.5 ± 10.7	0	100.0 ± 0.0	40.0 ± 41.8	53.3 ± 50.6
Session 5	10.5 ± 6.8	16.2 ± 20.0	1	80.0 ± 44.7	30.0 ± 27.4	25.0 ± 25.0
*A02*						
Session 4	2.5 ± 3.3	11.5 ± 12.5	0	100.0 ± 0.0	80.0 ± 27.4	40.7 ± 17.4
Session 5	13.9 ± 13.3	12.6 ± 6.7	0	100.0 ± 0.0	60.0 ± 41.8	63.3 ± 41.5
*A03*						
Session 4	9.2 ± 5.4	20.1 ± 11.8	0	100.0 ± 0.0	80.0 ± 27.4	58.0 ± 26.6
Session 5	2.5 ± 0.3	7.3 ± 6.4	0	100.0 ± 0.0	100.0 ± 0.0	32.0 ± 5.7
*A04*						
Session 4	2.7 ± 0.2	7.3 ± 2.6	0	100.0 ± 0.0	100.0 ± 0.0	31.4 ± 2.6
Session 5	9.5 ± 4.3	5.5 ± 2.1	1	100.0 ± 0.0	80.0 ± 27.4	22.7 ± 9.0
*A05*						
Session 4	9.7 ± 2.3	10.0 ± 3.2	0	100.0 ± 0.0	100.0 ± 0.0	47.3 ± 11.6
Session 5	8.6 ± 6.8	10.3 ± 4.5	0	100.0 ± 0.0	80.0 ± 27.4	32.0 ± 13.9

The outcomes of the MI questionnaire have been presented in Table 4. It is noteworthy that, with the exception of participant A02, all participants found visual MI to be a more accessible cognitive process compared to kinesthetic MI. The latter form of imagery appeared to require additional practice before achieving proficiency. However, only kinesthetic MI was employed in the experiments, as it is known to elicit comparable neural patterns to actual motor execution and has shown promise in facilitating rehabilitation processes [28].

Focusing on the specific scores attained within the kinesthetic MI category, participant A01 exhibited the highest level of difficulty in performing this particular form of imagery. These findings align with A01’s earlier calibration and closed-loop results, which indicated the lowest levels of proficiency.

**Table 4:** Results from MI questionnaire 3

	A01	A02	A03	A04	A05
Internal visual MI	4.5	7.0	6.5	6.3	6.3
External visual MI	5.5	7.0	6.8	6.5	7.0
Kinesthetic MI	3.3	7.0	5.3	5.8	6.5

## Discussion

Considering training data, deep learning approaches have outperformed the approach based on spatial features. Remarkably, the deep learning approach without fine-tuning achieved a performance level equivalent to that of the feature-based approach. This underscores the effectiveness of a generic model trained on data from different individuals and past sessions, attaining a comparable level of efficacy as the subject-specific and session-adapted feature-based approach. This observation holds significant implications for the reduction of calibration time, as it suggests that subjects may not need to perform training trials in every session while still achieving optimal performance.

On the other hand, with respect to the differences among the three deep learning approaches, the fine-tuning of all layer weights was the one that showed the highest performance. These findings are in line with our previous research [40]. However, differences were only significant for motion data. This provides evidence that models can still further benefit when they are adapted to each subject.

Subjects showed significant differences in success rates, both in calibration and closed-loop evaluation. It is something already presented in the literature [14, 36, 37]. In this research, MI questionnaire was used to see if those differences could be identified before using the system.

The MI questionnaire was proven to be an efficient tool to analyze the ability of each subject to use a BMI based on MI. In this research, although subjects experimented different levels of difficulty in performing imagery, there were not applied any differences in the experimental protocol. It could be interesting to include this test as a metric for inclusion/exclusion criteria or to define tailor-made training approaches that could benefit all subjects.

Regarding the comparison between open-loop and closed-loop trials, it is important to note that there is no direct correlation in terms of performance. Despite subject A04 achieved the highest decoding results in the open-loop phase, its performance did not translate to the best outcomes in closed-loop control. Conversely, subject A01 exhibited similar performance for both approaches. During real-control experiments, various factors come into play that can impact performance, which were absent during the calibration phase. Firstly, the mental state of the subject differs as they anticipate the reception of feedback, leading to a state of expectation. Additionally, their emotional state can be influenced by the results they obtain, leading to feelings of excitement or frustration. However, during the calibration phase they were unaware of their task performance [7, 41].

The outcomes from the open-loop phase reveal an accuracy that is comparatively lower than some other MI-based BMIs documented in the literature [6]. It is imperative to acknowledge the absence of a standardized method for evaluating MI performance across the existing literature. Many studies treat the entire duration of the MI task as a unified class, deeming a trial 100% accurate solely if MI is detected during that trial [6, 7]. In contrast, this study provides epoch-based metrics. It is also noteworthy that the methods proposed in this investigation are deployable in real-time, distinguishing them from more complex methodologies. Lastly, it is crucial to note that subjects received feedback exclusively during sessions 4 and 5 in the closed-loop control; consequently, their ability to adapt their activity was limited to the training phase of session 5.

The closed-loop trials employed in our experimental protocol aimed to simulate real-life scenarios, where participants had the autonomy to make decisions regarding actions such as walking or stopping gait. This approach allowed participants to have greater control over the situation, reducing potential biases compared to cue-based approaches that could negatively impact the results [14, 36, 42, 43]. It is challenging to directly compare the performance of our study with other works in the literature due to the adoption of different evaluation methodologies [6, 12].

The Static model demonstrated superior efficiency compared to the Motion model. In nearly all trials, the Static model successfully decoded subjects’ intentions to initiate gait, with only a single exception in subject A01 and fifth session. Conversely, the Motion model exhibited a higher susceptibility to errors, as indicated by a Ratio of Stops that never surpassed 65%. This implies that the exoskeleton halted the gait more frequently than the subjects’ actual intentions. To address this issue, novel paradigms have emerged that explore the identification of subject-perceived errors, which could potentially be incorporated to mitigate and increase this ratio [35].

### Limitations

This research has some limitations, especially regarding the dataset.The able-bodied participants in the research shared common characteristics, such as being in their twenties, demonstrating right-dominance laterality, and lacking prior experience with a BMI. While these shared features establish a baseline, it’s crucial to acknowledge the homogeneity of this participant group, potentially constraining the generalizability of our findings.

Furthermore, participants engaged in five sessions, yet feedback on their performance was only provided during sessions 4 and 5, specifically with the closed-loop control of the exoskeleton. Consequently, the limited exposure to the system might have constrained their ability to fully adapt or comprehend its usage.

Importantly, the envisioned users for this system are individuals with spinal cord injuries and motor limitations. Therefore, the inferences drawn from able-bodied participants may not accurately reflect the experiences or challenges faced by this target group.

Despite efforts made, there were notable variations in BMI performance among subjects, making it challenging to formulate definitive conclusions about system efficacy. Additionally, the dataset size, while aligning with typical participant numbers in many BMI applications [44], may not be extensive enough to encompass the full spectrum of potential usage outcomes. Consequently, determining the average efficiency of the system proves difficult. Subsequent research endeavors should prioritize expanding the sample size to enhance the representativeness of the study population and provide a more comprehensive understanding of the system’s performance across diverse users.

## Conclusion

The present study introduces a BMI utilizing MI practice to control a lower-limb exoskeleton. Participants underwent five experimental sessions, with the initial session aimed at familiarizing them with the system, two sessions to record brain activity during various mental tasks, and two sessions to evaluate the system performance.

Four different approaches were compared to decode brain signals and convert them into control commands. Three approaches utilized deep learning frameworks, while the fourth approach involved manually extracted features using CSP methodology. Among the deep learning approaches, two explored fine-tuning, where the model learned from data collected from other participants and sessions was adjusted to each subject and session. Results revealed that the deep learning algorithms achieved performance levels equal to or even surpassing that of CSP. Notably, the fully fine-tuned neural network yielded the highest performance, suggesting promising prospects for reducing calibration time. This deep learning approach was employed for evaluation in closed-loop control.

Furthermore, the evaluation protocol employed in this study simulated a real-life scenario where participants navigated through a pathway. This approach was deemed more intuitive for subjects, as it granted them the freedom to initiate each mental task. Future investigations in this field should consider incorporating such asynchronous control paradigms to further enhance the subject experience and system performance.

### Supplementary Information

**Additional file 1.** Inclusion and exclusion criteria It contains detailed information regarding the inclusion and exclusion criteria employed in participant selection for the experiments conducted. These criteria were established in accordance with the specifications provided by equipment manufacturers, ensuring precision and consistency in participant inclusion or exclusion.

## Data Availability

The datasets supporting the conclusions of this article are available in the Figshare repository, 10.6084/m9.figshare.24083994.

## Abbreviations

MI: Motor imagery
BMI: Brain–machine interface
fMRI: Functional magnetic resonance imaging
EEG: Electroencephalography
ECoG: Electrocorticography
PSD: Power spectral density
CSP: Common spatial patterns
EOG: Electrooculography
MIQ-3: Motor imagery questionnaire-3
CAR: Common average reference
LDA: Linear discriminant analysis
