# Supplementary material for: Brain–machine interface based on deep learning to control asynchronously a lower-limb robotic exoskeleton: a case-of-study
Source: J Neuroeng Rehabil. 2024 Apr 5;21:48. doi: 10.1186/s12984-024-01342-9 (PMC10996198; doi:10.1186/s12984-024-01342-9)
Supplement: Supplementary file 1 — Additional file 1. Inclusion and exclusion criteria It contains detailed information regarding the inclusion and exclusion criteria employed in participant selection for the experiments conducted. These criteria were established in accordance with the specifications provided by equipment manufacturers, ensuring precision and consistency in participant inclusion or exclusion. [file 12984_2024_1342_MOESM1_ESM.pdf]

Inclusion and exclusion criteria

Inclusion criteria for healthy individuals:

Participants will be recruited if they -
(1) age between 21-70 years.

(2) weight< 100kg.

(3) height between 160-190 cm.

(4) have intact, normal neurological and musculoskeletal functioning to perform the experimental protocols.

(5) are free of lower-body muscle and joint injuries or abnormalities.

(6) do not suffer from psychiatric illness or cognitive deficits that hinder learning.

(7) give informed consent.

(8) students can also participate if they are 21 years old or older.

Exclusion criteria for healthy individuals*:*

Potential participants will be excluded if they –
(1) have cognitive impairment.

(2) have uncontrolled epilepsy.

(3) have uncontrolled psychiatric illness.

(4) have tumors.

(5) have fractures.

(6) have history of osteoporosis (spine and hip z score < -2) .

(7) have heterotopic ossifications.

(8) have acute tendon or joint pathology of the upper or lower limbs.

(9) have severe lower limb dysmetria.

(10) have joint stiffness in joints of the lower limbs.

(11)have a cardiorespiratory pathology that limits their ability to exert themselves.

(12) have acute arterial or venous vascular disease.

(13) have orthostatic hypotension.

(14) have dermopathies. Pressure ulcers.

(15) have infections in general.

(16) are younger than 21 years of age or older than 70 years of age.

(17) are prisoners.

(18) are pregnant.

(19)height of the participant is not between 160-190 cm or the weight is larger than 220 lbs (100 Kg).
